# Human tumor viruses: induction of three-dimensional alterations in the host genome structure

**DOI:** 10.3389/fmicb.2023.1280210

**Published:** 2023-10-19

**Authors:** Janos Minarovits

**Affiliations:** Department of Oral Biology and Experimental Dental Research, Faculty of Dentistry, University of Szeged, Szeged, Hungary

**Keywords:** tumor virus, oncogene, transformation, chromatin loop, host cell genome, viral episome, integration

## Abstract

Certain viruses called tumor viruses or oncoviruses are capable to change the gene expression pattern of distinct human or animal cell types in tissue culture, resulting in uncontrolled proliferation as well as a change in the social behavior of the infected cells: the oncovirus-transformed, immortalized cells are capable to form malignant neoplasms in suitable animal models. At present, seven human viruses are categorized as causative agents of distinct human malignancies. The genomes of human tumor viruses, typically encode viral oncoproteins and non– translated viral RNAs that affect the gene expression pattern of their target cells or induce genetic and epigenetic alterations contributing to oncogenesis. Recently, the application of chromatin conformation capture technologies and three-dimensional (3D) molecular imaging techniques revealed how the gene products or genomes of certain human tumor viruses interact with and induce alterations in the 3D host genome structure. This Mini Review aims to cover selected aspects of these developments. The papers, discussed briefly, describe how insertion of a novel viral binding site for the 3D genome organizer cellular protein CCCTC-binding factor (CTCF) into the DNA of T cells infected by human T-cell lymphotropic virus type 1 (HTLV-1) may contribute to lymphomagenesis, as well as how integration of high risk human papillomavirus genome into the host cell DNA may facilitate cervical carcinogenesis. Recent results regarding the interactions of cellular genomes with the episomal, chromatinized DNA genomes of oncogenic human herpesvirus, Epstein–Barr virus (EBV) will also be summarized, similarly to available data regarding contacts formed by episomal or integrated hepatitis B virus (HBV) DNA with host chromatin. Finally, a putative mechanism of hepatitis C virus (HCV) induced chromatin alterations will be presented, which may solve the riddle, how a cytoplasmic RNA virus without a viral oncogene could induce malingnant transfrormation of hepatocytes.

## 1. Introduction

Application of novel sequencing-based and microscopy-based techniques allowed high-resolution mapping of chromatin contacts and permitted three-dimensional reconstruction of nuclear architecture in various cell types (Lieberman-Aiden et al., 2009; Jerkovic and Cavalli, 2021). Using a refined version (*in situ* Hi.C) of the sequencing-based chromosome conformation capture (3C) method, Rao et al. mapped chromatin interactions, at a 1 Kb resolution, in a human B lymphoblastoid cell line immortalized with Epstein–Barr virus (Rao et al., 2014). Similar interaction maps were created in 8 other human and mouse cell lines, allowing the identification of six distinct nuclear subcompartments displaying characteristic patterns of histone modifications, and approximately 10, 000 chromatin loops that may allow long-range enhancer-promoter interactions (see reference 3, for a detailed characterization of nuclear subcompartments). Recently, a single chromatin loop situated in the vicinity of the *TCRA* (*T Cell Receptor A*) locus in human chromosome 14 could be visualized by Parteka-Tojek et al., using a combination of FISH staining and interferometric photoactivated localization microscopy (iPALM) (Parteka-Tojek et al., 2022). They used the very same EBV-immortalized human lymphoblastoid cell line (GM12878) that was characterized by Rao et al., who mapped that particular loop by *in situ* Hi-C (Rao et al., 2014). Parteka-Tojek et al. concluded that the physical distance maps generated by single-cell imaging fitted well to the population based genomic data (Parteka-Tojek et al., 2022).

Chromatin loops are formed by the interaction of a ring-shaped protein complex called cohesin and the nuclear protein CCCTC-binding factor (CTCF); reviewed by Beagan and Phillips-Cremins (2020).

Approximately 10% of cancer cases is attributable to virus infections (Plummer et al., 2016). Analysis of whole-genome and whole-transcriptome sequencing data of cancer specimens revealed a high prevalence of certain human tumor-viruses, including Epstein–Barr virus (EBV), hepatitis B virus (HBV) and high risk human papilloma viruses (HPV16 and HPV18) (Zapatka et al., 2020). These viruses carry a **DNA** genome in their virion.

EBV, a human gammaherpesvirus, infects both lymphoid and epithelial cells, and it is regarded as the causative agent of B lymphoproloferative disease; its cell tropism is reflected in its association with Hodgkin lymphoma, Burkitt lymphoma and diffuse large B cell lymphoma (Shannon-Lowe and Rickinson, 2019). In addition, EBV is associated with T/NK lymphoproliferative diseases and T/NK lymphomas/leukemias, as well as with neoplasms of epithelial origin, nasopharyngeal carcinoma and a fraction of gastric carcinomas (Shannon-Lowe and Rickinson, 2019). In rare cases, leiomyosarcomas of smooth muscle origin also carry EBV genomes (Shannon-Lowe and Rickinson, 2019; Zhang et al., 2023).

HBV is one of the causative agent of hepatocellular carcinoma; upon infection of liver cells, the partially double-stranded **DNA** genome carried by the HBV virion is converted to a covalently closed, circular DNA (cccDNA) in the host cell nucleus (Seeger and Mason, 2015). Chronic HBV infections frequently progress to cirrhosis, a risk factor for developing liver cancer (D’souza et al., 2020).

So called high risk human papilloma viruses (HPV16, HPV18) are regarded as causative agents of genital cancers, especially cervical carcinoma; the genomes of these **DNA** tumor viruses encode oncoproteins interacting with so called tumor suppressor proteins that regulate cell proliferation (Zur Hausen, 2009; Pal and Kundu, 2020).

In addition to EBV, HBV, and HPV, two other human viruses that carry a **double-stranded DNA genome** in the virion, were also implicated in tumorigenesis, albeit they cause neoplasms of lower prevalence: Kaposi’s sarcoma-associated herpesvirus (KSHV, also called Human gammaherpesvirus 8, HHV-8) is associated with Kaposi’s sarcoma and primary effusion lymphoma, whereas the genome of Merkel cell polyomavirus(MCPyV) was frequently detected in cells of a rare, but aggressive malignancy, Merkel cell carcinoma (Mesri et al., 2010; Chakraborty et al., 2012; Czech-Sioli et al., 2020; Pietropaolo et al., 2020).

In contrast to the human tumor viruses listed above, Hepatitis C virus (HCV), a single stranded **RNA virus**, does not carry a viral oncogene in its genome (Yamagishi et al., 2018; Zaki et al., 2022). HCV is considered to be the causative agent of hepatocellular carcinoma (HCC) developing in individuals with chronic HCV infection (Yamagishi et al., 2018; Zaki et al., 2022). Both direct, viral associated, and indirect, immune-associated factors contribute to the development of HCV-associated liver cancer (Zaki et al., 2022). Table 1 summarizes the human tumor viruses and their target cells. The genomes of human tumor viruses typically carry genes coding for viral oncoproteins implicated in tumorigenesis (Vogt, 2012; Chang et al., 2017; Gaglia and Munger, 2018; Krump and You, 2018; Stolz and McCormick, 2020). Oncoproteins frequently target tumor suppressor pathways or host signaling pathways that regulate cell proliferation (Krump and You, 2018). Certain RNA tumor viruses infecting animals do not carry, however, viral oncogenes; they may cause malignant tumors by inserting a DNA copy of their RNA genome into the host cell DNA and activating cellular genes that control cell proliferation *via* the inserted viral promoter or enhancer sequences; see (Robinson and Gagnon, 1986), and references therein. It was suggested that a similar mechanism of insertional tumorigenesis is involved in the initiation and progression of HTLV-1-associated T-cell leukemia-lymphoma as well, as discussed below (see **2**.). Thereafter, I wish to summarize how integrated HPV DNA and episomal HPV-host cell DNA hybrid sequences may affect the 3D structure of host cell chromatin (see **3**.). Latent, episomal EBV genomes also interact with the host cell chromatin in both lymphoid and epithelial cells (see **4**.), whereas extrachromosomal, episomal HBV genomes contact active regions of target cell chromatin at an early stage of infection, followed by integration and loop formation at a later stage (see **5**.), Unexpectedly, it was also observed that HCV, an RNA tumor virus that does not enter the host cell nucleus and does not carry a viral oncogene, may cause misregulation of cohesin, a nuclear protein regulating the 3D genome structure, contributing thereby to a change of host cell gene expression and development of liver cancer (see **6.**). Finally, I would like to outline briefly how **a new research field**, focusing on tumor virus induced alterations in the 3D host genome, may open new possibilities for diagnostics and therapy viral as well as non-viral malignancies (see **7**.).

**Table 1:** Human tumor viruses and their targets.

Virus family and name	Genome (in the virion)	Target cells
Herpesviridae		
Epstein–Barr virus (EBV)	DNA	lymphoid, epithelial & smooth muscle cells
Kaposi’s sarcoma-associated herpesvirus (KSHV)	DNA	endothelial & lymphoid cells
Papillomaviridae		
Human papillomavirus (HPV)	DNA	epithelial cells
Polyomaviridae		
Merkel cell polyomavirus (MCPyV)	DNA	neuroectodermal cells
Hepadnaviridae		
Hepatitis B virus (HBV)	DNA	liver cells
Retroviridae		
Human T-lymphotropic virus, type 1 (HTLV-1)	RNA	T cells
Flaviviridae		
Hepatitis C virus (HCV)	RNA	liver cells

As discussed in this Mini Review, EBV, HPV, HBV, HTLV-I, and HCV are capable to induce alterations in the 3D structure of host cell genomes. Further studies may reveal whether similar mechanisms contribute to oncogenesis by KSHV and MCPyV, too.

## 2. Insertion of a novel, viral CTCF binding site carried by HTLV-I provirus into the host cell genome may rewire chromatin loops in infected T cells

Using CTCF Chip assays, Satou et al. observed that CTCF, a regulator of chromatin structure, bound to the so called pX region of HTLV-1 provirus in a nonmalignant HTLV-1 infected T cell clone and in a T cell line derived from adult T cell leukemia (ATL) (Satou et al., 2016). Furthermore, similar observations were made using fresh PBMCs (peripheral blood mononuclear cells) from a patient with HTLV-1–associated myelopathy/tropical spastic paraparesis (HAM/TSP), a chronic inflammatory disease, and PBMCs of a patient with ATL, indicating that CTCF binds to the HTLV-1 provirus *in vivo* as well (Satou et al., 2016). They compared long-range chromatin interactions in cells carrying either wild type HTLV-I provirus or a mutant HTLV-1 provirus (ΔvCTCF-BS) unable to associate with the zinc finger protein CTCF and demonstrated by quantitative chromosome conformation capture (3C) analysis a decreased interaction frequency in case of the mutant provirus (Satou et al., 2016). In a follow-up study.Melamed et al. applied the method of quantitative 4C (q4C, a modified version of circular chromosome conformation capture) to analyse chromatin contacts formed between HTLV-1 genomes integrated into the DNA of CD4+ T-cell clones, and the relevatnt host cell genome (Melamed et al., 2018). The T-cell clones studied were isolated from HTLV-1-infected individuals, and long-range chromatin contacts could be detected between the HTLV-1provirus and the host genome in 9 of the 10 clones, in parallel with alterations in host gene expression (Melamed et al., 2018). Although these experiments suggested that insertional mutagenesis contributes to ATL pathogenesis, Martinez et al. found that the viral CTCF binding site was dispensable for T-cell immortalization and for persistent infection *in vivo* (Martinez et al., 2019). The situation is complex, because HTLV-1-encoded oncoproteins also contribute to ATL induction, and in infected individuals the provirus is present at unique integration sites in a series of T-cell clones. One may speculate that distinct CTCF-CTCF contacts, formed between integrated HTLV-I genomes and cellular chromatin loops alter the cellular transcriptome in a manner that provides growth advantage for selected T cell clones during ATL initiation or progression.

## 3. Integration of human papillomavirus (HPV) DNA into the host cell genome: reprogramming of host cell transcription *via de novo* generation of a cellular super-enhancer

So called “high risk” human papillomaviruses, especially HPV type 16 and 18 (HPV16, HPV18) are the causative agents of cervical cancer. Although their circular, double stranded DNA genomes typically persist as extrachromosomal episomes in the nuclei of infected epithelial cells, cervical carcinoma cells usually carry integrated HPV16 and HPV18 DNA sequences in their genome. Genetic and epigenetic consequences of integration result in enhanced expression of the viral oncogenes *E6* and *E7,* as reviewed in (McBride and Warburton, 2017). Warburton et al. observed that multiple, tandemly integrated HPV16 genomes in chromosome 2 of the cervical-derived cell line 20,861 formed a so called super-enhancer (SE) with co-amplified cellular sequences (Warburton et al., 2018). Accordingly, this locus was enriched in Bromodomain-containing protein 4 (BRD4), a regulator of transcription, and in acetylated histone H3 (H3K27ac), another marker of super-enhancers that ensure enhanced expression of distal genes *via* chromatin looping. As a matter of fact, this particular locus directed high level expression of a viral-cellular fusion transcript encoding the HPV oncoproteins E6 and E7 (Warburton et al., 2018). Recently, Tian et al. characterized the sites of HPV integration in HPV-positive cell lines and observed that HPV integration frequently resulted in the generation of cellular super-enhancers called HPV breakpoint-induced cellular SEs (BP-cSEs) (Tian et al., 2023). Furthermore, they showed that some of the BP-cSEs were formed by extrachromosomal, HPV DNA-human DNA hybrid sequences and in the case of HeLa cells they could even trace back the origin of three extrachromosomal DNAs (ecDNAS) to the integration sites of HPV18 genomes on chromosome 8. These sequencing data supported the idea that the hybrid ecDNAs were induced, indeed, by the integration of viral genomes into the host DNA (Tian et al., 2023). Hi-C, Chip-seq and RNA seq data revealed that the episomal, ecDNA based super-enhancers could establish long-range intra- and extra-chromosomal interactions and acted as transcriptional regulators (Tian et al., 2023).

## 4. Latent Epstein–Barr virus genomes interact with and change the 3D structure of host cell genomes

Latent, growth-transformation associated genes of Epstein–Barr virus (EBV), an oncogenic human herpesvirus, are typically expressed from circular, chromatinized viral episomes in host cell nuclei (Shannon-Lowe and Rickinson, 2019). Although it is well documented that host-cell dependent expression of EBV-encoded latent proteins and non-translated RNAs contrbutes to tumorigenesis, recent studies revealed that direct interactions of the viral episomes with the cellular genome may also play a role in the induction of EBV-associated malignancies. Kim et al. studied Burkitt lymphoma (BL) cell lines using circular chromosome conformation capture (4C), and observed that the EBV episomes preferably associated with intergenic regions of the cellular genome (Kim et al., 2020). They compared the BL data with the results of Rao et al. (2014) who studied an EBV-immortalized B lymphoblastoid cell line (LCL), and found that the EBV genomes formed more contacts with promoter rich regions in the latter (Kim et al., 2020). A re-analysis of public data sets by Wang et al., as well as their own 4C-seq data (4C-seq: circular chromatin conformation capture followed by deep sequencing) supported the view that EBV episomes colocalise with active regions of the host chromatin in LCLs (Wang et al., 2020). I would like to add that besides episome-host genome interactions, EBV-encoded oncoproteins, especially nuclear antigens also significantly contribute to the reorganization of host cell chromatin during immortalization of resting B cells and establishment of LCLs *in vitro* (Wang et al., 2023).

In addition to lymphoid malignancies, EBV is also associated with certain neoplasms of epithelial origin, including a subset of gastric carcinomas. Okabe et al. found that in EBV-positive gastric carcinoma cell lines the viral episomes form long-range interactions with distinct chromatin domains of the host cell, accompanied by heterochromatin-to euchromatin transitions (Okabe et al., 2020). Okabe et al. suggested that such interactions, called ‘enhancer infestation’, may induce epigenetic alterations and activate cellular oncogenes (proto-onc genes), contributing to carcinogenesis (Okabe et al., 2020).

## 5. Chromatin reorganization in hepatitis B virus (HBV) infected hepatoma cells

Using Hi-C sequencing, Guo et al. analysed chromatin interactions in a HBV-infected human hepatoma cell line and its uninfected counterpart (Guo et al., 2023). They observed enhanced chromatin interactions, especially on distinct interphase chromosomes, in HBV-positive cells and recorded characteristic shifts in nuclear subcompartments, accompanied with repression of enhancers regulating inflammatory genes and opening of regions enriched in transposable elements (Guo et al., 2023). These data suggested that reorganization of 3D genome structure may contribute to disease development associated with HBV infection. Yang et al. studied two *in vitro* HBV infection systems corresponding to a *de novo* infection stage with episomal viral genomes and a later stage with integrated HBV genomes (Yang et al., 2020). HBV DNA-host DNA contacts were assessed using 3C-high-throughput genome-wide translocation sequencing (3C-HTGTS), and the results showed that at the initial stage of infection the HBV minichromosome (covalently closed circular DNA, cccDNA) preferably contacts transcriptionally active regions of host cell chromatin, whereas the integrated HBV genome, especially when integrated to a transcriptionally active region on chromosome 2, interacted with the host genome and formed a chromatin loop structure (Yang et al., 2020).

## 6. Hepatitis C virus (HCV) up-regulates the RAD21 cohesin subunit, a regulator of chromatin structure: implications for hepatocarcinogenesis induced by a cytoplasmic RNA virus

Hepatitis C virus (HCV) is one of the causative agents of hepatocellular carcinoma, its exact role, however, in carcinogenesis is unknown at present (Goossens and Hoshida, 2015). HCV has a single stranded RNA genome, but lacks a viral oncogene, and in contrast to retroviruses that replicate *via* a DNA intermediate integrating into the host cell genome, HCV replication is confined to the cytoplasm. Perhaps for this reason, until recently the interaction of HCV with the host cell chromatin happened to be outside the focus of the research community. As mentioned in the Introduction, chromatin loops are formed by the interaction of a ring-shaped protein complex called **c**ohesin and the nuclear protein CCCTC-binding factor (CTCF) (Beagan and Phillips-Cremins, 2020). Perez et al. studied hepatoma cells and primary hepatocytes infected by hepatitis C virus(HCV) *in vitro*, and observed an increased expression of RAD21, a core component of the cohesin complex, in HCV-infected cells (Perez et al., 2019). In parallel, they documented that cohesin binding sites were enriched at regulator elements of the cellular genome in HCV-infected cells, and there were changes in their transcriptome as well, compared to uninfected controls. Furthermore, HCV also induced centrosome abnormalities, possibly due to cohesin misregulation, Based on preliminary data, Perez et al. argued that NS3/4A, a HCV-encoded protease, may degrade the cohesin regulator WAPL (Wings apart-like protein homolog) protein in HCV-infected cells, resulting in dysregulation of gene expression and induction of chromosomal aberrations (Perez et al., 2019).

## 7. Perspectives

Tumor viruses or oncoviruses that show a strong association with various neoplastic diseases in humans belong to several virus families (Table 1). Their replication strategies and target cells are heterogenous, and as explored by intense research efforts, a variety of molecular mechanisms contribute to the generation of malignant tumors by oncovirus infected cells. These research efforts had important implications for other areas as well, including cell biology, tumor biology, non-viral carcinogenesis and oncotherapy. As reflected in this Mini Review, the study of tumor virus induced alterations in the 3D host genome is a booming, novel research field that resulted in new concepts and ideas as to the mechanism of malignant transformation, similarly to the studies dealing with changes of the 2D host genome elicited by retroviral insertional mutagenesis (Bushman, 2020). Exploring how tumor viruses affect the 3D structure of host cell gnomes may open new possibilities for molecular-targeted therapy of virus-associated neoplastic diseases, and may have implications for the therapy of non-viral malignancies, too.

## Author contributions

JM: Conceptualization, Writing – original draft, Writing – review & editing.
